# Supplementary material for: Direct Pattern Growth of Carbon Nanomaterials by Laser Scribing on Spin-Coated Cu-PI Composite Films and Their Gas Sensor Application
Source: Materials (Basel). 2021 Jun 18;14(12):3388. doi: 10.3390/ma14123388 (PMC8235262; doi:10.3390/ma14123388)
Supplement: Supplementary file 1 [file materials-14-03388-s001.zip › Suppelemtary-materials-1265823/Supplementary materials-1265823 .pdf]

Supplementary Materials

Direct pattern growth of carbon nanomaterials by laser scribing on spin-coated Cu-PI composite films and their gas sensor application

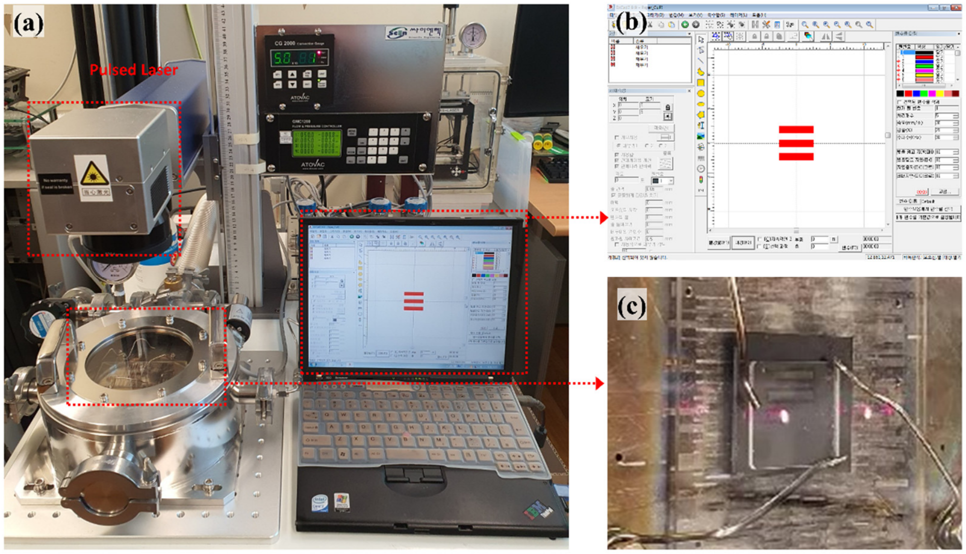

Figure S1. Digital photographs of (**a**) laser-assisted CVD system, (**b**) operating software and (**c**) target sample in chamber (see the "Laser assisted CVD system.mp4" video file).

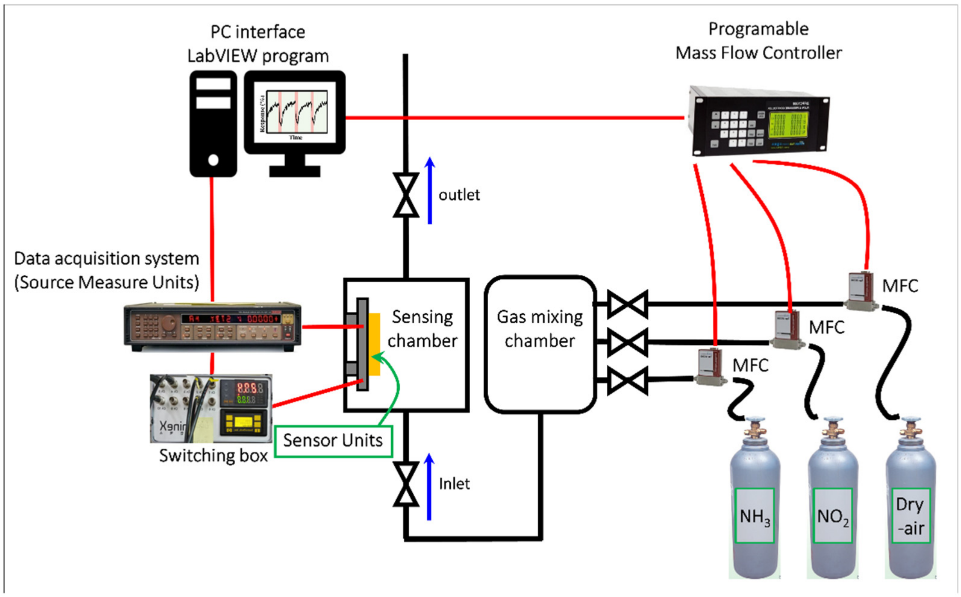

Figure S2. Schematic diagram of gas sensing system.

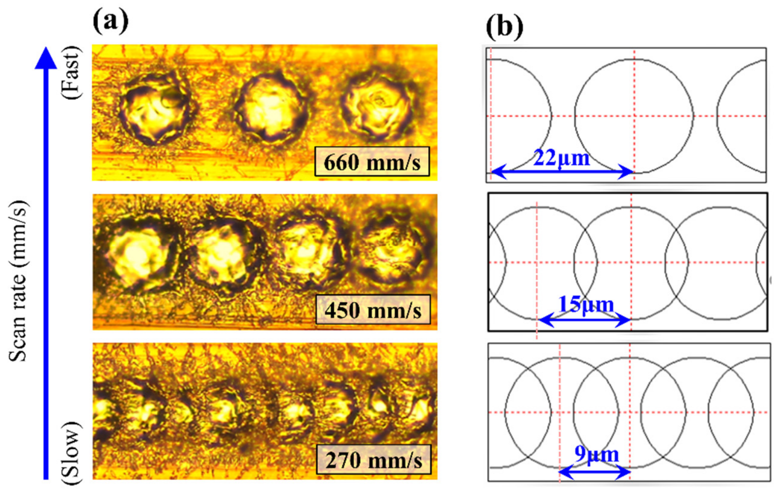

Figure S3. Optical images of pulsed-laser scribed spots onto the copper foil with the (**a**) different scan rate and (**b**) schematic of spot distance.

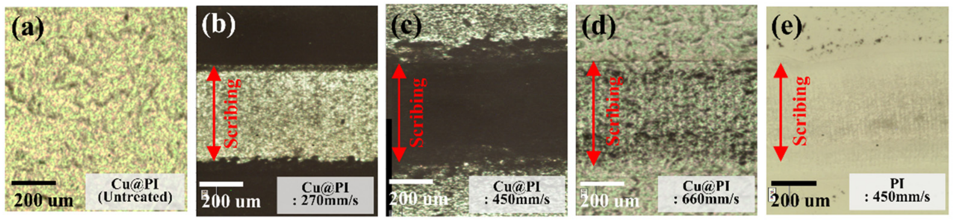

Figure S4. Optical images of (**a**) pristine Cu@PI, (**b**-**d**) Cu@LSC after laser scribing with different scan rate, and (**e**) LSC without copper.

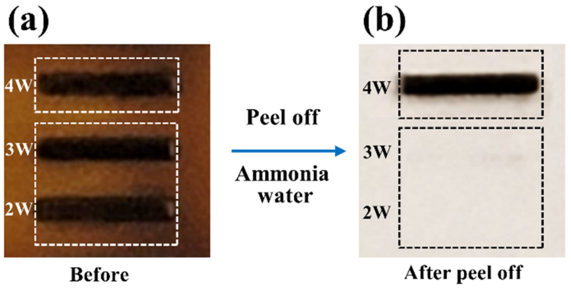

Figure S5. Photographs of Cu@LSC (**a**) before and (**b**) after peel off the untreated layer by dipping in ammonia water.

Table S1. Specific parameters of laser scriber (MFP-20).

Parameter	Wavelength (nm)	Average output power (W)	Energy/Pulse (mj)	Beam diameter (μm)	Freqyency range (kHz)
Data	1064 ± 4	20	0.8~1.0	6~9	30~60
**Parameter**	**Pulse width (ns)**	**Power range (%)**	**Operating voltage (V)**	**Power consumption (W)**	**Scan speed (mm/s)**
Data	80~140	5~100	24	170	0~1500

Table S2. Specific synthesis conditions of the Cu@LSC with.

I.D	Cu/PI ratio (wt.%)	Spin coating (rpm)	Trans-mit-tance (%)	Power (W)	Scan rate (mm/s)	Spot Distance (um)	I_D/I_G	I_{2D}/I_G
A	2.5	1000	75.3	4	450	15	1.57	n/a
B	5.0	1000	57.6	4	450	15	0.95	0.16
C	7.5	1000	36.1	4	450	15	0.57	0.69
D	10.0	1000	21.9	4	450	15	0.52	0.71
E	10.0	1000	21.9	4	450	15	0.52	0.71
F	10.0	1500	40.3	4	450	15	0.57	0.48
G	10.0	2000	63.5	4	450	15	0.88	0.34
H	10.0	2500	81.4	4	450	15	1.05	0.25
I	10.0	1000	21.9	4	270	9	n/a	n/a
J	10.0	1000	21.9	4	450	15	0.52	0.71
K	10.0	1000	21.9	4	660	22	n/a	n/a
L	10.0	1000	21.9	2	450	15	n/a	n/a
M	10.0	1000	21.9	3	450	15	n/a	n/a
N	10.0	1000	21.9	4	450	15	0.52	0.71
O	10.0	1000	21.9	5	450	15	0.85	0.18
